# Case–control study of paternal occupational exposures and childhood lymphoma in Great Britain, 1962–2010

**DOI:** 10.1038/s41416-019-0469-7

**Published:** 2019-05-20

**Authors:** Kathryn J. Bunch, Gerald M. Kendall, Charles A. Stiller, Timothy J. Vincent, Michael F. G. Murphy

**Affiliations:** 1 National Perinatal Epidemiology Unit, Nuffield Department of Population Health, University of Oxford, Richard Doll Building, Old Road Campus, Oxford, OX3 7LF UK; 2 Cancer Epidemiology Unit, Nuffield Department of Population Health, University of Oxford, Richard Doll Building, Old Road Campus, Oxford, OX3 7LF UK; 3 National Cancer Registration and Analysis Service, Public Health England, Chancellor Court, Oxford Business Park South, Oxford, OX4 2GX UK; 4 Formerly of Childhood Cancer Research Group, University of Oxford, Oxford, UK; 5 Nuffield Department of Women’s and Reproductive Health John Radcliffe Hospital, University of Oxford, Oxford, OX3 9DU UK

**Keywords:** Cancer epidemiology, Risk factors

## Abstract

**Background:**

This nationwide study investigates associations between paternal occupational exposure and childhood lymphoma.

**Methods:**

The UK National Registry of Childhood Tumours provided cases of childhood lymphoma born and diagnosed in Great Britain 1962–2010. Control births, unaffected by childhood cancer, were matched on sex, birth period and birth registration sub-district. Fathers’ occupations were assigned to one or more of 33 exposure groups and also coded for occupational social class.

**Results:**

We analysed 5033 childhood lymphoma cases and 4990 controls. Total lymphoma and the subgroups Hodgkin, Burkitt and non-Hodgkin lymphoma were considered separately. No one exposure was significantly associated with increased risk within all subgroups and for total lymphoma. However, exposure to “ceramics and glass” was significantly associated with increased risk of total lymphoma, Hodgkin and non-Hodgkin lymphoma. Paternal lead exposure was associated with Burkitt lymphoma and exposure to metal fumes was associated with Hodgkin lymphoma.

**Conclusions:**

This study provides no support for previous suggestions of an association between childhood lymphoma and paternal occupational exposure to pesticides, solvents/hydrocarbons or infections potentially transmitted by father’s social contacts. An association with exposure to “ceramics and glass” was noted for the two major lymphoma subtypes together comprising 80% of total lymphoma.

## Background

The UK National Registry of Childhood Tumours (UK NRCT) holds a substantially complete record of childhood cancers diagnosed in Great Britain 1962–2010 together with birth registrations for these case children and matched controls.^1^ Several papers based on these data have been published investigating associations between individual cancer subtypes (retinoblastoma,^2^ Wilms tumour,^3^ neuroblastoma,^4^ leukaemia^5^ and central nervous system tumours^6^) and inferred paternal occupational exposures to potential risk factors. This paper continues the series by investigating incidence of childhood lymphomas from the UK NRCT.

Lymphoma is the third most common childhood cancer in Great Britain and northern Europe, accounting for nearly 10% of new cases diagnosed in those aged <15 years.^7–9^ In Great Britain, about 140 children developed lymphoma each year in the period 1991–2000 with incidence tending to increase with calendar year.^7^ Lymphoma accounts for a similar proportion of childhood cancer in most regions of the world^10^ with the exceptions of North and sub-Saharan Africa where it is relatively more prevalent. The two largest subgroups Hodgkin lymphoma (HL) and non-Hodgkin lymphoma (NHL), defined in the International Classification of Childhood Cancer version 3 (ICCC-3)^11^ as ICCC-3 21 and 22, respectively, each account for around 60 cases a year. The third most numerous subgroup, Burkitt lymphoma (BL; ICCC-3 23), contributes nearly 20 cases each year. While some researchers combine BL with other NHL, we have considered them separately. Two small lymphoma categories, Miscellaneous lymphoreticular neoplasms (ICCC-3 24) and Unspecified lymphomas (ICCC-3 25), contribute few cases (~200 and 100 in total, respectively, over the entire period 1962–2010) and were included only in our analysis of total lymphoma.

Childhood lymphoma is markedly more common in boys than in girls and incidence rates increase with age for both HL and NHL, while peak incidence occurs between 5 and 9 years of age for BL. Unlike leukaemia, there is no evidence for an association between birth weight and childhood lymphoma.^12,13^

## Aetiology of lymphoma

Roman and Smith remark^14^ that epidemiological reports on lymphomas often begin, and sometimes end, by stating that little is known about the causes of the condition under study. However, infection has been implicated in some types of lymphoma. Such a connection is clear in the case of BL^15,16^ and very probable also for HL.^15^ However, a more complex aetiology is suggested for other NHL.^17^ For childhood HL, it has been proposed that an infectious aetiology may result in socio-economic status (SES) variations caused by delayed infection in more affluent groups.^18^ Both Greaves and Kinlen have advanced hypotheses suggesting an infectious aetiology for some childhood cancers. Greaves’s “delayed infection hypothesis” relates specifically to acute lymphoblastic leukaemia.^19^ Kinlen has proposed a “Population Mixing Hypothesis” under which some childhood leukaemia is a rare response to an as yet unidentified infection.^20^ Kinlen has published extensive data to support this hypothesis and in at least one instance the effect extends specifically to NHL.^21^

In addition to infection, ionising radiation is another known risk factor for childhood cancer, in particular, leukaemia.^22^ Because of diagnostic uncertainties in the 1950s and 1960s, childhood NHL has often been combined with leukaemia (and termed LNHL) particularly in studies of clusters of these diseases around nuclear installations.^23^ However, opinion now inclines towards a mechanism involving infection as an explanation for these clusters^24^ and there is certainly no suggestion that they are driven by radiation-induced NHL. UNSCEAR^25,26^ concluded that, while data were sparse, radiation had not been found to be associated with the development of lymphomas.

## Occupational exposure of parents

The risk of cancer in adults from exposures experienced at work has been widely studied, perhaps most notably by the International Agency for Research on Cancer (IARC).^27^ IARC produces a series of monographs on known and suspected human carcinogens that has recently been updated.^28,29^ Occupational exposures in the UK have also been reviewed.^30,31^ Population-based studies of occupational disease often make use of job–exposure matrices to infer probable exposures from standard occupational classifications.^32^ There has also been concern that occupational exposures at work may be risk factors for cancer in the children of workers. Savitz and Chen,^33^ reviewing the literature up to 1989, reported that a number of studies had found associations between childhood leukaemia/lymphoma and parental occupations involving motor vehicle-related occupations or those involving exposures to paints and pigments. However, none of these studies had investigated lymphomas alone, and it appeared that the focus was on leukaemia. Colt and Blair^34^ updating this review to 1997 again found no evidence of associations between paternal occupation and childhood lymphoma. Again, however, the focus was on leukaemia.

More recent studies specifically focussed on lymphoma have been published. McKinney et al.^35^ reported statistically significant associations between the risk of childhood NHL and maternal exposure to solvents. In a study by Miligi et al.,^36^ the risk of NHL appeared to be related to paternal exposure to oxygenated solvents and petrol exhausts. Pearce et al.^37^ reported significant associations between father’s occupations that involved social contact and NHL when controls from the Cumbrian Births database were used; when Registry controls were used, the association was positive but not significant. Olsen et al.^38^ reported significantly elevated numbers of cancers in children of fathers employed in the manufacture of iron and metal structures, in machine repair workshops and as machinists and smiths and that there might be particular associations with lymphomas. However, they did not give numerical results.

There remains a longstanding interest in the possibility of a link between pesticide exposure and childhood cancer, including lymphoma. Zahm and Ward^39^ conducted a systematic review of published studies of general pesticide exposure and childhood cancer, but parental occupational exposure was only one possible contribution. They concluded that there was evidence linking NHL and pesticide exposure, though interpretation was limited by nonspecific pesticide exposure information, small numbers of exposed subjects and the potential for case-response bias. Infante-Rivard and Weichenthal^40^ updated the review of Zahm and Ward with a further 21 studies and concluded that a number of epidemiological studies consistently reported increased associations between pesticide exposures and NHL, but a causal relationship was still not demonstrated.

Previous studies have thus provided some suggestions of links between parental exposure to a wide variety of agents and childhood lymphoma. Many are likely to be chance findings and none emerges as a likely strong carcinogen. Perhaps the most consistent associations have been with exposures to hydrocarbons and other solvents and to pesticides. In addition, there are indications of a role for infections, suggesting that offspring of parents who have high levels of social contact might be at risk. However, there is no convincing evidence that these are strong risk factors.

The present study investigates associations between paternal occupational exposure and childhood lymphoma in Great Britain using data from birth registration records for cases and controls held by the UK NRCT 1962–2010 within a matched case–control study design. We focus on paternal occupation because it is more completely recorded on birth registrations during our study period than maternal occupation.^41^ This large, nationwide study addresses some of the shortcomings of previous studies, particularly low numbers of cases and the risks of selection and recall bias.^42^ We also use job title to derive an approximate measure of paternal social class to investigate the possible independent associations between SES and childhood lymphoma.

## Methods

### Cases and controls

Five thousand eight hundred and seventy-five registered cases of lymphoma in children aged <15 years born and diagnosed between 1962 and 2010 in Britain were identified from the UK NRCT. A total of 272 cases were excluded because they were born overseas or adopted. A further 189 cases for whom no birth registration could be found were also excluded, leaving 5414 eligible cases for whom a birth record was available.

Control children were selected from birth registers, held by the Office for National Statistics (ONS) or the General Register Office for Scotland (GROS). For this study, one control for each case was used, matched on sex, period of birth and birth registration sub-district.

The completeness of ascertainment of childhood cancer cases in the NRCT has varied over time, but it contains a substantially (>97%) complete record of all childhood cancers registered in Britain from the early 1970s.^7,43^

Oxfordshire Research Ethics Committee (Oxfordshire REC C, Ref 12/SC/0532) approved the use of these data in 2012.

### Coding of occupational groups

In the UK, paternal occupation is routinely recorded on the public record of birth registrations where the father is named. Paternal occupation was abstracted verbatim from the case and control birth records as supplied by ONS and GROS.

Occupations were coded according to the 1980 Office of Population Censuses and Surveys (OPCS) Classification of Occupations.^44^ Coding was carried out independently by two coders using the OPCS (now ONS) coding manuals. Where the two coders disagreed, a third coded the occupation. Where the third coder agreed with one of the original coders, that agreed code was assigned. Where all three coders disagreed, the occupation was coded as “uncodable”. At all stages, occupations were coded blind to the case–control status of the individuals.

For 317 cases and 367 controls, paternal occupation was missing and these subjects were excluded from the analysis. For some (46 cases and 36 controls), it was not possible to assign an occupation code, or it was not possible to convert the 1980 code to a 1970 code (18 cases and 21 controls). In these circumstances, the paternal occupation was coded as missing.

### Coding of occupational exposure groups

This made use of a job–exposure matrix that had been developed by Fear et al.^41^ using occupational classifications from the 1970 Classification of Occupations.^45^ The 1980 classifications were accordingly converted to the 1970 scheme using bridge codes.^41,46^ The 1970 codes were subsequently allocated to one or more of the 33 occupational exposure groups, which had previously been associated with cancer or with adverse reproductive outcomes in the offspring of men exposed to them. The assignments were on the basis of specialist experience, examination of job descriptions and literature concerning occupational exposures.^41^ These job–exposure associations have been described in detail elsewhere.^41,47^ Occupations not appearing in any of the 33 groups were classified “unexposed” in all groups.

Occupations classified to one or more of the exposure groups were further defined as having either “definite” (daily contact with the agent or contact at a high intensity) or “possible” (exposure to the agent neither daily nor at high intensity) exposure in that group. Job titles could be coded to more than one occupational exposure group, for example, bus drivers appear as exposed in “exhaust fumes”, “inhaled hydrocarbons” and “social contact”.

Some exposures could be incurred in combination with others. Details and discussion are given in the Supplementary Material on Exposures.

### Coding of occupational social class

Each 1980 occupation code was then assigned to one of the six social class codes from the 1980 OPCS Classification of Occupations.^48^ For 496 cases and 559 controls, social class was classified as “missing” because no occupation was given or the occupation falls outside the ONS social classifications (i.e. armed forces, student, independent means or sick). The 18 cases and 21 controls excluded from the occupation analysis as described above were included in the social class analysis and appear in the results shown in Table 2.

Two hundred and seven case and 229 control fathers were classified as “forces”, comprising the armed forces, police force, fire service and guards and related workers not elsewhere classified. Within the “forces” group, social class code was unavailable for members of the armed forces, approximately half the group, and so the adjusted analysis was not performed for this exposure group.

### Analysis

Odds ratios (ORs) and 95% confidence intervals (95% CIs) for our matched analysis were calculated using conditional logistic regression.^49^ Matching factors were: sex, period of birth, and birth registration sub-district. ORs and 95% CIs additionally adjusted for social class (I, II, IIINM, IIIM, IV and V) were also generated. Our primary exposed population was those classified as “definitely” exposed. The same analyses were repeated taking the exposed population as those with either “definite” or “possible” exposures. Differences between these two sets of results were minimal and all further references are to definite exposures. In instances where there were ≤5 exposed cases and/or controls for any analysis, exact conditional logistic regression was used. Because of the small numbers of cases/controls, adjustment for social class was inappropriate in these cases. Statistically significant results were defined as those where the *p* value was <0.05 and any significant ORs reflecting associations not previously reported in the literature were re-assessed using the Bonferroni method^50^ to allow judgements to be made on the importance of multiple significance testing. In these circumstances, simple *p* values are likely to suggest significance for associations that are simply due to chance. However, the Bonferroni correction is likely to fail to identify genuinely significant associations. We suggest that further information and in particular additional independent studies are required to resolve such ambiguities.

All statistical analyses were carried out using STATA 11.^51^ Results are shown as forest plots in the main text. In these figures, data for exposures with <=5 cases and/or controls have been suppressed. However, full numerical results are in Supplementary Material on Detailed Results.

## Results

Supplementary Table B1 gives details of the records excluded at various stages of setting up the set of cases and controls for analyses. After exclusions, a total of 5033 (93%) cases and 4990 (92%) controls were included in the unadjusted analyses of occupation and lymphoma risk and 4918 (91%) cases and 4855 (90%) controls in analyses of social class and lymphoma risk. Of the included cases, 2003 (40%) were HL, 2257 (45%) were NHL and 484 (10%) were BL (Table 1). There was little difference in social class between the cases and controls.

**Table 1:** Characteristics of lymphoma cases and their cancer-free controls born and diagnosed in Great Britain between 1962 and 2010 for whom a birth record and ONS occupation code were available

	Cases		Controls	
	Number	Percent	Number	Percent
Sex				
Male	3467	69	3437	69
Female	1566	31	1553	31
Total	5033	100	4990	100
Lymphoma subtype				
Hodgkin lymphoma	2003	40	1981	40
Non-Hodgkin lymphoma	2257	45	2254	45
Burkitt lymphoma	484	10	465	9
Misc. lymphoreticular neoplasms	184	4	189	4
Unspecified lymphoma	105	2	101	2
Total	5033	100	4990	100
Birth decade				
1962–1969	1174	23	1173	23
1970–1979	1179	23	1184	24
1980–1989	1243	25	1219	24
1990–1999	1168	23	1142	23
2000–2010	269	5	272	5
Total	5033	100	4990	100
Occupational social class				
I	363	7	355	7
II	1013	20	959	19
IIINM	547	11	616	12
IIIM	1811	36	1768	35
IV	860	17	849	17
V	306	6	287	6
Not known	133	3	156	3
Total	5033	100	4990	100
Region				
North	302	6	290	6
Yorkshire and Humberside	487	10	483	10
East Midlands	361	7	356	7
East Anglia	150	3	149	3
South East	1657	33	1665	33
South West	352	7	352	7
West Midlands	496	10	487	10
North West	578	11	546	11
Wales	230	5	241	5
Scotland	416	8	419	8
Not known	4	0	2	0
Total	5033	100	4990	100

*M* Manual, *NM* Non-Manual, *ONS* Office for National Statistics

The 5033 cases had occupations with which a total of 5904 exposures were associated. The number of exposures per case varied from zero to five (Supplementary Table A1). The pattern for controls was broadly similar. About 36% of cases and controls had occupations with which none of the selected exposures were associated and about 34% had a single exposure; the remaining 30% had between 2 and 5 exposures. Details are given in Supplementary Table A2.

**Fig. 1:**
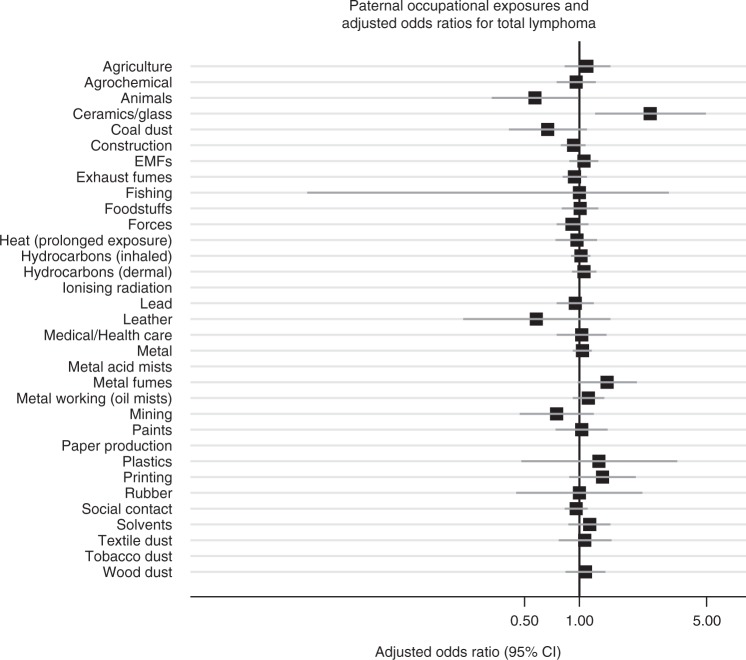
Forest plot of adjusted odds ratios for total lymphoma and paternal occupational exposures

Figure 1 and Supplementary Table B2 show estimates for the risk of total lymphoma by occupational exposure group. Lymphoma risk was borderline significantly reduced (OR 0.58, 95% CI 0.34–1.00) in the children of fathers exposed to animals, although this effect ceased to be significant on adjustment for occupational social class. Paternal exposure to ceramics and/or glass resulted in a significantly raised lymphoma risk both before and after adjustment for social class (OR 2.33, 1.19–4.59 and 2.45, 1.22–4.95, respectively), although after Bonferroni adjustment for multiple testing, these raised ORs ceased to be significant.

**Fig. 2:**
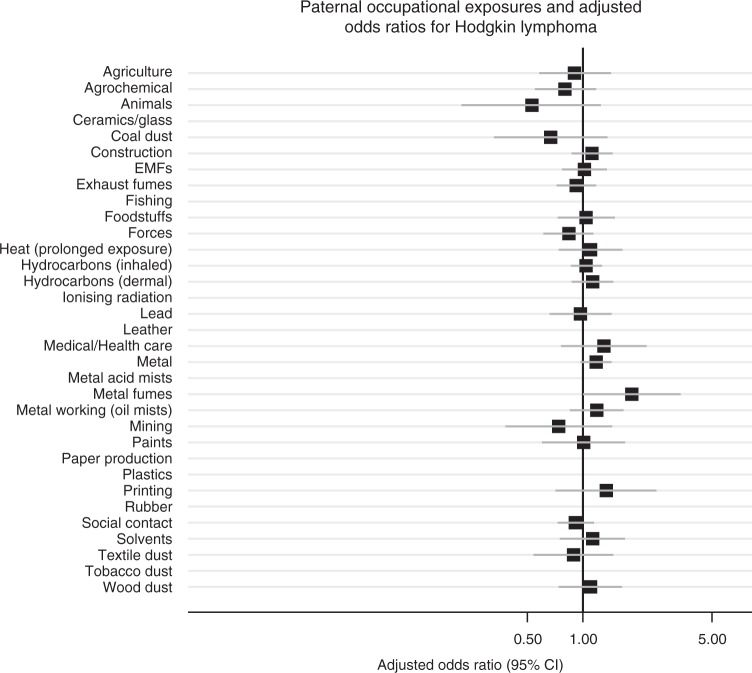
Forest plot of adjusted odds ratios for Hodgkin lymphoma and paternal occupational exposures

**Fig. 3:**
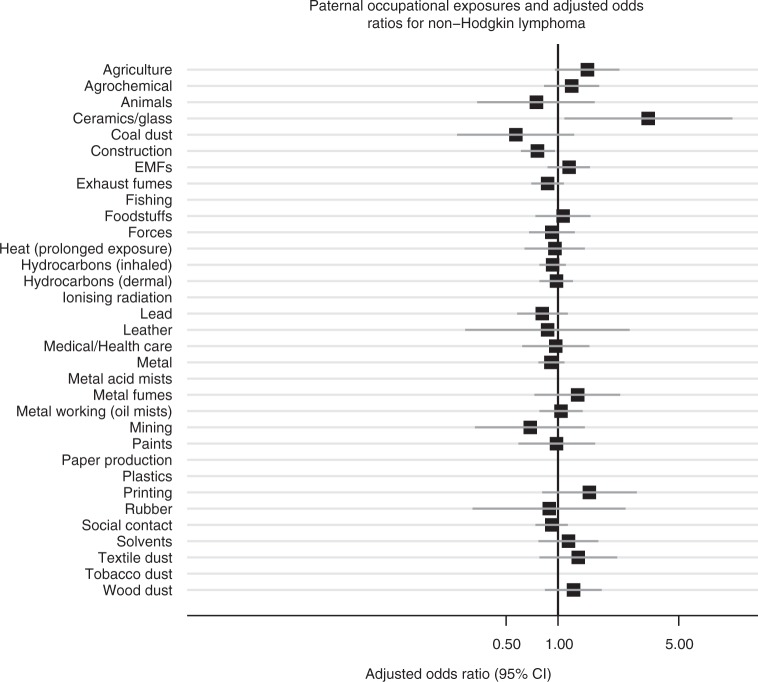
Forest plot of adjusted odds ratios for non-Hodgkin lymphoma and paternal occupational exposures

**Fig. 4:**
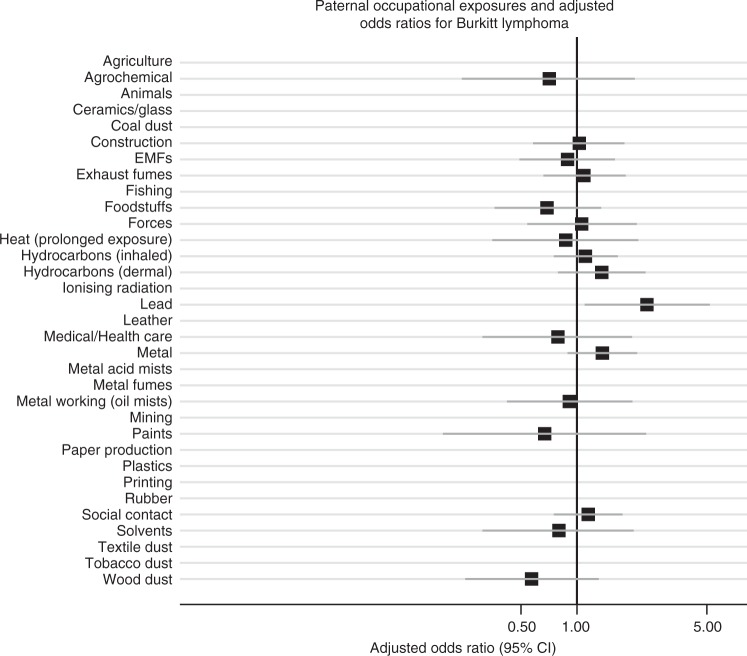
Forest plot of adjusted odds ratios for Burkitt lymphoma and paternal occupational exposures

Considering the lymphoma subgroups separately (Figs. 2, 3, 4 and Supplementary Tables B3, B4, B5), paternal exposure to ceramics and/or glass was associated with a significantly raised risk for both HL and NHL (OR 4.00, 1.08–22.09 and 2.80, 1.01–7.77, respectively) and remained so after adjustment for occupational social class for both subgroups. HL risk was also positively associated with paternal exposure to metal fumes (OR 1.88, 1.02–3.44) with adjustment for social class only slightly reducing the risk. Paternal employment in construction resulted in a significantly reduced risk for NHL (OR 0.77, 0.62–0.97) with little change on adjustment. BL risk was significantly raised on paternal exposure to lead (OR 2.67, 1.24–5.74) and remained so after adjustment. However, all these individual raised risks ceased to be significant after adjusting for multiple testing.

The exposures most consistently associated with childhood lymphoma in previous studies were pesticides (agrochemicals, exposure group 1), infections (social contact, group 29) and hydrocarbons/solvents (groups 13, 14, 30). In no case were ORs in the present study significantly elevated for these exposure groups, whether for all lymphomas combined or for the subgroups.

Table 2 gives a breakdown of lymphoma risk by occupational social class. ORs were calculated relative to class III Manual. For lymphoma as a whole, the OR was significantly lower for class III Non-Manual and higher for class IV. But there were no significant differences for classes I, II or V and no significant trend.

**Table 2:** Lymphoma risk by paternal occupationally defined social class

Social class of the father	Controls	Hodgkin lymphoma		Non-Hodgkin lymphoma		Burkitt lymphoma		Total lymphoma	
		Cases	OR^a^ (95% CI)	Cases	OR^a^ (95% CI)	Cases	OR^a^ (95% CI)	Cases	OR^a^ (95% CI)
I	355	142	0.89 (0.59–1.36)	171	0.83 (0.56–1.24)	32	0.55 (0.20–1.47)	363	0.81 (0.62–1.06)
II	959	397	1.05 (0.82–1.35)	454	1.01 (0.80–1.28)	117	0.93 (0.54–1.60)	1014	1.02 (0.87–1.20)
III Non-Manual	616	206	**0.66 (0.47–0.91)**	260	0.92 (0.68–1.25)	52	0.52 (0.25–1.09)	547	**0.76** **(0.62–0.93)**
III Manual	1770	738	1.00	806	1.00	159	1.00	1813	1.00
IV	849	340	1.05 (0.80–1.36)	379	1.25 (0.99–1.59)	81	1.44 (0.79–2.63)	860	**1.19 (1.01–1.40)**
V	306	133	0.87 (0.57–1.33)	145	1.06 (0.72–1.55)	26	2.33 (0.90–6.07)	321	1.07 (0.83–1.39)
Trend analysis^b^	4855		1.02 (0.97–1.07)		0.97 (0.93–1.02)		1.07 (0.96–1.19)	4918	1.01 (0.97–1.03)

The table includes 18 cases and 21 controls who had a social class code assigned but who had no occupation code assigned and were excluded from the occupation analysis. ORs in bold are significant *P* < 0.05, in bold and single underlined *P* < 0.01

*CI* confidence interval, *OR* odds ratio

^a^OR for the indicated ONS Social Class(es) with III Manual taken as the reference category

^b^OR for each increase in occupational social class

## Discussion

The aetiology of childhood lymphoma is little understood. This may be largely a consequence of the rarity of the disease and the consequent low numbers in epidemiological studies coupled perhaps with the absence of clearly identified and strongly associated causal agents. The literature briefly summarised in the introduction must be viewed with caution, chance findings and reporting and other biases being particular potential problems.^52^ However, more than one previous study has suggested associations between lymphomas and parental social contact (taken as a surrogate for infections), exposure to pesticides and exposure to solvents, petrol or work with motor vehicles. In our data set, we found no significant associations between lymphoma risk and any of these exposures.

We found significantly raised lymphoma risk both before and after adjustment for social class in the offspring of fathers exposed to ceramics and glass. Similar elevations were seen for both HL and NHL separately. In a population-based study of occupation and lymphoma in adults, glass formers and potters had the highest OR of any occupational group, though this was based on small numbers of cases and controls.^53^ We are not aware of other reports in the literature of associations between these occupational exposures and childhood lymphoma.

The IARC has reviewed exposures in the glass industry^54^ and concluded that some were probably carcinogenic to humans. However, they reported no convincing links between paternal exposure and childhood cancer. A substantial complication in the interpretation of studies in this area is the large number of potentially suspect materials involved in production. These include a wide variety of raw materials and “almost every metal in the periodic table” as colourants or for other purposes.^54^ Manufacturing involves high temperatures that will lead to the dispersal of volatile materials. IARC reported few published data on exposures in the glass industry, almost all of what there was related to lead and other metals.

Our study covered cases diagnosed between 1962 and 2010, a period of nearly 50 years, during which time it is highly likely that manufacturing processes and consequent levels of exposure to potential carcinogens changed. Breaking down the data into two shorter time periods suggested that the increased risk for total lymphoma was greatest in the period 1990–2010, but with only 15 case–control pairs informing this result, it should be treated with great caution. Similarly, our attempt to explore the raised risk for HL among children whose fathers had been exposed to metal fumes was hampered by small numbers of informative case–control pairs though there was perhaps weak evidence that this effect was greater during the first two decades of the study. It could perhaps be that health and safety regulations and protective equipment were more effective later in the period covered by the study. In particular, the Health and Safety at Work Act of 1974 may have been influential.

In principle, exposure to multiple agents might be a complication of our analysis. However, of the 43 individuals in occupations likely to be exposed to “ceramics and glass”, only 5 (3 cases and 2 controls) were exposed to more than one agent. These were furnace- or kiln-men in the ceramics and glass industry who were also likely to be exposed to prolonged heat and to hydrocarbons. We doubt that these exposures affected our findings.

We found no significant variations in lymphoma incidence with SES. This may be contrasted with the marked trends found for childhood leukaemia in Great Britain, both in a study analogous to the present one^5^ and in one using the Carstairs measure of SES, which is based on census data for the area rather than paternal occupation.^55^

### Strengths and limitations

The strengths of this study are that the analysis is based on approximately 5000 cases with data drawn from the UK NRCT, which has, over the period studied here, consistently high levels of case ascertainment.^43^ Problems with interview-based case–control studies are recall and participation bias. Such biases are very unlikely to arise in the present study since routinely collected data were used and occupation was documented before diagnosis. The exposure assessment used a well-established occupational and exposure classification,^41^ to which father’s occupation was coded blind to case–control status.

However, our method used self-reported occupation recorded at the time of birth, and this might differ from an occupation held at another, possibly more aetiologically important, time period. Additionally, our exposure categories are based on generic judgements; we have no specific information on the frequency or duration of exposure. Occupational practices and exposures may also have changed during the long study period that could lead to exposure misclassification.

Diagnostic practice and terminology also changed during the study period. BL, which was originally described in tropical Africa, had been recognised in non-endemic form in Britain by the mid-1960s.^56^ However, it was severely under-recorded in the early part of the study period, and some pathologists were still reporting it as lymphoblastic lymphoma in the 1990s.^57^ The age-standardised registration rate for childhood BL increased 20-fold from 0.10 per million in 1966–1970 to 2.11 per million in 1996–2000 [1], and it seems highly unlikely that this increase would have taken place uniformly across the country. Therefore, given that occupational exposures are bound to have varied geographically and over time, the results pertaining to BL should be treated with particular caution.

### Interpretation

One possible reason why our data have not shown associations between paternal occupational exposures and lymphoma risk is exposure misclassification. The exposure windows when a paternal occupational exposure may plausibly lead to childhood cancer are at peri-conception, as a result of effects of the exposure on germ cells, and during pregnancy and after birth, when contaminants brought home from the workplace by the father may affect the embryo or young child.^58,59^ Since we have no information about paternal occupation before or after a child’s birth was registered, the occupation (and hence exposure) may have been different and exposure misclassification may have arisen as a result. However, this applies equally to cases and controls. In addition, we have no direct information about the intensity or frequency of exposure within groups, and over the almost 50 years for which we have data, actual exposures may have changed within exposure groups as a result of changing workplace practices. Schuz el al. have noted^60^ the desirability of detailed information on the intensity and frequency of exposures.

## Conclusion

We conducted a large case–control study of childhood lymphoma and paternal occupational exposure to potential carcinogens. The study was record based and participation and recall bias are unlikely. Paternal occupational exposure to “ceramics and glass” appeared to be significantly associated with total lymphoma and both the major subgroups (HL and NHL), both before and after adjustment for occupational social class as a measure of the general SES of the family at the child’s birth. These associations ceased to be significant after allowing for the effects of multiple testing using the Bonferroni method. It will be important to see whether other studies confirm such an association. However, we note that such paternal exposures are unlikely to be common. In our series, <0.4% of all occupational exposures were to “ceramics and glass”.

Our results do not support previous suggestions of a role for exposure to pesticides, solvents/hydrocarbons or infections as transmitted via the father’s occupation in the aetiology of childhood lymphoma.

Perhaps the main weaknesses of the study are that the exposures are based on self-reported paternal occupation at the time of child’s birth, which may or may not be the most aetiologically important period; the assignment of exposures was generic and unchanging over the study period.

## Supplementary information

Supplementary Material

## Data Availability

The data are contained within the National Registry of Childhood Tumours.
