# Supplementary material for: Case–control study of paternal occupational exposures and childhood lymphoma in Great Britain, 1962–2010
Source: Br J Cancer. 2019 May 20;120(12):1153–61. doi: 10.1038/s41416-019-0469-7 (PMC6738046; doi:10.1038/s41416-019-0469-7)
Supplement: Supplementary file 1 — Supplementary Material [file 41416_2019_469_MOESM1_ESM.doc]

**Supplementary Material**

Supplementary Information on Exposures

Supplementary Table A1 - Number of exposures per study subject

Supplementary Table A2 - Number of exposures to each of the agents by Case-Control status

Supplementary Table A3 - Overlapping exposures in study subjects

Supplementary Information on Detailed Results

Supplementary Table B1 - Numbers of case and Control records in different categories for birth registrations and coding of occupation and social class

Supplementary Table B2 - Paternal Occupational Exposures and Odd Ratios for total lymphoma

Supplementary Table B3 - Paternal Occupational Exposures and Odds Ratios for Hodgkin lymphoma

Supplementary Table B4 - Paternal Occupational Exposures and Odds Ratios for non-Hodgkin lymphoma

Supplementary Table B5 - Paternal Occupational Exposures and ORs for Burkitt lymphoma

**Supplementary Material on Exposures to Potentially Hazardous Agents in Specific occupations**

In this paper we investigate the possible effects of paternal exposure to potentially hazardous agents on the risk of lymphoma in their offspring. Thirty three agents had been identified as having previously been associated with cancer or with adverse reproductive outcomes in the offspring of men exposed to them. Occupations likely to be associated with these exposures have been identified in published literature [1, 2].

We determined the occupations of our cases and of controls from the paternal occupation as shown on the birth certificate of the child in question. We chose to study paternal occupations because, for our study period (1962-2010) the corresponding data for mothers were very much less complete. In this on-line Appendix we provide extra detail of these occupational exposures too bulky for the main text. In what follows the reader should remember that the father’s occupation determines the agents to which he was (likely to be) exposed.

Table 1 shows the exposures associated with the various categories of employment of the fathers of the study subjects. There were between 0 and 5 exposures per occupation and the relative numbers of exposures for controls were similar to those for cases. Just over a third of study subjects had fathers in occupations associated with none of the selected agents and roughly the same proportion with a single agent. The remainder were likely to be exposed to two or more (up to five) agents

The occupations with 5 exposures were

19 Rolling, tube mill operators, metal drawers (4 controls)

41 Motor mechanics, auto engineers (79 controls; 85 cases)

85 Compositors (6 controls; 10 cases)

Exposures to a given agent might occur in more than one occupation. Thus “Social Contact” was likely in 36 occupations and “Inhaled hydrocarbons” in 30.

Table 2 shows the number of cases and controls likely to be exposed to each of the agents under study. Because some occupations are associated with exposures to more than one agent, in many cases these exposures occurred in combination rather than singly. Table 2 gives, for cases and controls together, the numbers of exposures incurred on their own and the number incurred in combination.

Table 3 gives more details of the way that exposures overlap. In this table all overlaps on or below the leading diagonal have been omitted for clarity. In the first line it is shown that all 226 exposures to “agriculture” also involve exposure to “agrochemical”. Line 2 shows that 76 (302-226) study subjects were exposed to “agrochemicals” but not to “agriculture”, for example in the occupational group Foresters and Woodmen. The 60 study subjects exposed to “animals” were mostly (59) butchers, also exposed to “foodstuffs” and one tanner who was also exposed to “leather” and to “solvents”. The 43 study subjects exposed to “ceramics and glass” were mostly (39) exposed to no other agent, but four furnacemen were also exposed to prolonged heat and to hydrocarbons, both inhaled and dermal.

It is clear that such exposures to more than one agent must be borne in mind when interpreting any positive finding. This is discussed in the main text.

References

1. Fear N, Roman E, Reeves G*, et al.* Father's occupation and childhood mortality: analysis of routinely collected data. Health statistics quarterly 1999;2:7-15.

2. Fear NT, Roman E, Reeves G*, et al.* Are the children of fathers whose jobs involve contact with many people at an increased risk of leukaemia? Occup Environ Med 1999;56(7):438-442.

Associated Tables:

Table A1: Number of exposures per study subject

Table A2: Number of exposures to each of the agents by Case/Control status.

Table A3: Overlapping exposures in study subjects

Table A1: Number of exposures per study subject

|  | Cases | | | Controls | | | Total | | |
| --- | --- | --- | --- | --- | --- | --- | --- | --- | --- |
| Number of Exposures | Frequency | Percent | Total exposures | Frequency | Percent | Total exposures | Frequency | Percent | Total exposures |
| 0 | 1821 | 36.2 | 0 | 1,772 | 35.5 | 0 | 3593 | 35.8 | 0 |
| 1 | 1690 | 33.6 | 1690 | 1,726 | 34.6 | 1726 | 3416 | 34.1 | 3416 |
| 2 | 892 | 17.7 | 1784 | 910 | 18.2 | 1820 | 1802 | 18.0 | 3604 |
| 3 | 185 | 3.7 | 555 | 203 | 4.1 | 609 | 388 | 3.9 | 1164 |
| 4 | 350 | 7.0 | 1400 | 290 | 5.8 | 1160 | 640 | 6.4 | 2560 |
| 5 | 95 | 1.9 | 475 | 89 | 1.8 | 445 | 184 | 1.8 | 920 |
| Total | 5033 |  | 5904 | 4,990 |  | 5760 | 10023 |  | 11664 |
| Mean |  |  | 1.17 |  |  | 1.15 |  |  | 1.16 |

Table A2: Number of exposures to each of the agents by Case/Control status.

Also given are the number of exposures associated with no other and with some other agents ("Single" and "Multiple" respectively)

|  | ***Exposure*** | ***Controls*** | ***Cases*** | ***Total*** | ***Single*** | ***Multiple*** |
| --- | --- | --- | --- | --- | --- | --- |
| 1 | Agriculture | 107 | 119 | 226 | 0 | 226 |
| 2 | Agrochemical | 152 | 150 | 302 | 76 | 226 |
| 3 | Animals | 38 | 22 | 60 | 0 | 60 |
| 4 | Ceramics/glass | 14 | 29 | 43 | 38 | 5 |
| 5 | Coal dust | 47 | 34 | 81 | 0 | 81 |
| 6 | Construction | 393 | 374 | 767 | 767 | 0 |
| 7 | EMFs | 265 | 281 | 546 | 436 | 110 |
| 8 | Exhaust fumes | 432 | 423 | 855 | 11 | 844 |
| 9 | Fishing | 10 | 8 | 18 | 18 | 0 |
| 10 | Foodstuffs | 170 | 169 | 339 | 100 | 239 |
| 11 | Forces | 229 | 207 | 436 | 214 | 222 |
| 12 | Heat (prolonged exposure) | 129 | 127 | 256 | 0 | 256 |
| 13 | Hydrocarbons (inhaled) | 823 | 859 | 1682 | 18 | 1664 |
| 14 | Hydrocarbons (dermal) | 416 | 446 | 862 | 56 | 806 |
| 15 | Ionising radiation | 4 | 5 | 9 | 5 | 4 |
| 16 | Lead | 167 | 157 | 324 | 0 | 324 |
| 17 | Leather | 14 | 8 | 22 | 21 | 1 |
| 18 | Medical/health care | 95 | 94 | 189 | 12 | 177 |
| 19 | Metal | 757 | 790 | 1547 | 620 | 927 |
| 20 | Metal acid mists | 4 | 4 | 8 | 0 | 8 |
| 21 | Metal fumes | 59 | 78 | 137 | 0 | 137 |
| 22 | Metal working (oil mists) | 216 | 241 | 457 | 0 | 457 |
| 23 | Mining | 48 | 38 | 86 | 5 | 81 |
| 24 | Paints | 82 | 93 | 175 | 0 | 175 |
| 25 | Paper production | 1 | 1 | 2 | 2 | 0 |
| 26 | Plastics | 8 | 11 | 19 | 19 | 0 |
| 27 | Printing | 45 | 58 | 103 | 0 | 103 |
| 28 | Rubber | 12 | 13 | 25 | 0 | 25 |
| 29 | Social contact | 665 | 661 | 1326 | 538 | 788 |
| 30 | Solvents | 138 | 164 | 302 | 0 | 302 |
| 31 | Textile dust | 79 | 86 | 165 | 165 | 0 |
| 32 | Tobacco dust | 0 | 1 | 1 | 1 | 0 |
| 33 | Wood dust | 141 | 153 | 294 | 294 | 0 |
| 34 | Total | 5760 | 5904 | 11664 | 3416 | 8248 |

Table A3: Overlapping exposures in study subjects

|  |  |  | 1 | 2 | 3 | 4 | 5 | 6 | 7 | 8 | 9 | 10 | 11 | 12 | 13 | 14 | 15 | 16 | 17 | 18 | 19 | 20 | 21 | 22 | 23 | 24 | 25 | 26 | 27 | 28 | 29 | 30 | 31 | 32 | 33 |
| --- | --- | --- | --- | --- | --- | --- | --- | --- | --- | --- | --- | --- | --- | --- | --- | --- | --- | --- | --- | --- | --- | --- | --- | --- | --- | --- | --- | --- | --- | --- | --- | --- | --- | --- | --- |
|  |  | Total number (cases/controls) exposed | Agriculture | Agrochemical | Animals | Ceramics/glass | Coal dust | Construction | EMFs | Exhaust fumes | Fishing | Foodstuffs | Forces | Heat (prolonged exposure) | Hydrocarbons (inhaled) | Hydrocarbons (dermal) | Ionising radiation | Lead | Leather | Medical/Health care | Metal | Metal acid mists | Metal fumes | Metal working (oil mists) | Mining | Paints | Paper production | Plastics | Printing | Rubber | Social contact | Solvents | Textile dust | Tobacco dust | Wood dust |
|  | Total number (cases/controls) exposed |  | 226 | 302 | 60 | 43 | 81 | 767 | 546 | 855 | 18 | 339 | 436 | 256 | 1682 | 862 | 9 | 324 | 22 | 189 | 1547 | 8 | 137 | 457 | 86 | 175 | 2 | 19 | 103 | 25 | 1326 | 302 | 165 | 1 | 294 |
| 1 | Agriculture | 226 |  | 226 |  |  |  |  |  |  |  |  |  |  |  |  |  |  |  |  |  |  |  |  |  |  |  |  |  |  |  |  |  |  |  |
| 2 | Agrochemical | 302 |  |  |  |  |  |  |  |  |  |  |  |  |  |  |  |  |  |  |  |  |  |  |  |  |  |  |  |  |  |  |  |  |  |
| 3 | Animals | 60 |  |  |  |  |  |  |  |  |  | 59 |  |  |  |  |  |  | 1 |  |  |  |  |  |  |  |  |  |  |  |  | 1 |  |  |  |
| 4 | Ceramics/glass | 43 |  |  |  |  |  |  |  |  |  |  |  | 5 | 5 | 5 |  |  |  |  |  |  |  |  |  |  |  |  |  |  |  |  |  |  |  |
| 5 | Coal dust | 81 |  |  |  |  |  |  |  |  |  |  |  |  | 43 |  |  |  |  |  |  |  |  |  | 81 |  |  |  |  |  |  |  |  |  |  |
| 6 | Construction | 767 |  |  |  |  |  |  |  |  |  |  |  |  |  |  |  |  |  |  |  |  |  |  |  |  |  |  |  |  |  |  |  |  |  |
| 7 | EMFs | 546 |  |  |  |  |  |  |  |  |  |  |  | 98 |  |  | 4 |  |  | 4 | 106 | 8 |  | 98 |  |  |  |  |  |  |  |  |  |  |  |
| 8 | Exhaust fumes | 855 |  |  |  |  |  |  |  |  |  |  |  |  | 738 | 164 |  | 164 |  |  | 164 |  |  |  |  |  |  |  |  |  | 286 |  |  |  |  |
| 9 | Fishing | 18 |  |  |  |  |  |  |  |  |  |  |  |  |  |  |  |  |  |  |  |  |  |  |  |  |  |  |  |  |  |  |  |  |  |
| 10 | Foodstuffs | 339 |  |  |  |  |  |  |  |  |  |  |  |  | 99 |  |  |  |  |  |  |  |  |  |  |  |  |  |  |  | 81 |  |  |  |  |
| 11 | Forces | 436 |  |  |  |  |  |  |  |  |  |  |  | 35 | 35 |  |  |  |  |  |  |  |  |  |  |  |  |  |  |  | 187 |  |  |  |  |
| 12 | Heat (prolonged exposure) | 256 |  |  |  |  |  |  |  |  |  |  |  |  | 158 | 73 |  |  |  |  | 152 |  | 137 | 4 |  |  |  |  |  |  |  |  |  |  |  |
| 13 | Hydrocarbons (inhaled) | 1682 |  |  |  |  |  |  |  |  |  |  |  |  |  | 779 |  | 180 |  |  | 650 |  | 39 | 436 | 43 |  |  |  | 103 | 25 | 218 | 103 |  |  |  |
| 14 | Hydrocarbons (dermal) | 862 |  |  |  |  |  |  |  |  |  |  |  |  |  |  |  | 180 |  |  | 627 |  |  | 457 |  |  |  |  | 82 |  |  | 82 |  |  |  |
| 15 | Ionising radiation | 9 |  |  |  |  |  |  |  |  |  |  |  |  |  |  |  |  |  | 4 |  |  |  |  |  |  |  |  |  |  |  |  |  |  |  |
| 16 | Lead | 324 |  |  |  |  |  |  |  |  |  |  |  |  |  |  |  |  |  |  | 308 |  |  |  |  |  |  |  | 16 |  |  | 16 |  |  |  |
| 17 | Leather | 22 |  |  |  |  |  |  |  |  |  |  |  |  |  |  |  |  |  |  |  |  |  |  |  |  |  |  |  |  |  | 1 |  |  |  |
| 18 | Medical/Health care | 189 |  |  |  |  |  |  |  |  |  |  |  |  |  |  |  |  |  |  |  |  |  |  |  |  |  |  |  |  | 173 |  |  |  |  |
| 19 | Metal | 1547 |  |  |  |  |  |  |  |  |  |  |  |  |  |  |  |  |  |  |  | 8 | 137 | 457 |  |  |  |  |  |  |  |  |  |  |  |
| 20 | Metal acid mists | 8 |  |  |  |  |  |  |  |  |  |  |  |  |  |  |  |  |  |  |  |  |  |  |  |  |  |  |  |  |  |  |  |  |  |
| 21 | Metal fumes | 137 |  |  |  |  |  |  |  |  |  |  |  |  |  |  |  |  |  |  |  |  |  |  |  |  |  |  |  |  |  |  |  |  |  |
| 22 | Metal working (oil mists) | 457 |  |  |  |  |  |  |  |  |  |  |  |  |  |  |  |  |  |  |  |  |  |  |  |  |  |  |  |  |  |  |  |  |  |
| 23 | Mining | 86 |  |  |  |  |  |  |  |  |  |  |  |  |  |  |  |  |  |  |  |  |  |  |  |  |  |  |  |  |  |  |  |  |  |
| 24 | Paints | 175 |  |  |  |  |  |  |  |  |  |  |  |  |  |  |  |  |  |  |  |  |  |  |  |  |  |  |  |  |  | 175 |  |  |  |
| 25 | Paper production | 2 |  |  |  |  |  |  |  |  |  |  |  |  |  |  |  |  |  |  |  |  |  |  |  |  |  |  |  |  |  |  |  |  |  |
| 26 | Plastics | 19 |  |  |  |  |  |  |  |  |  |  |  |  |  |  |  |  |  |  |  |  |  |  |  |  |  |  |  |  |  |  |  |  |  |
| 27 | Printing | 103 |  |  |  |  |  |  |  |  |  |  |  |  |  |  |  |  |  |  |  |  |  |  |  |  |  |  |  |  |  | 103 |  |  |  |
| 28 | Rubber | 25 |  |  |  |  |  |  |  |  |  |  |  |  |  |  |  |  |  |  |  |  |  |  |  |  |  |  |  |  |  |  |  |  |  |
| 29 | Social contact | 1326 |  |  |  |  |  |  |  |  |  |  |  |  |  |  |  |  |  |  |  |  |  |  |  |  |  |  |  |  |  | 23 |  |  |  |
| 30 | Solvents | 302 |  |  |  |  |  |  |  |  |  |  |  |  |  |  |  |  |  |  |  |  |  |  |  |  |  |  |  |  |  |  |  |  |  |
| 31 | Textile dust | 165 |  |  |  |  |  |  |  |  |  |  |  |  |  |  |  |  |  |  |  |  |  |  |  |  |  |  |  |  |  |  |  |  |  |
| 32 | Tobacco dust | 1 |  |  |  |  |  |  |  |  |  |  |  |  |  |  |  |  |  |  |  |  |  |  |  |  |  |  |  |  |  |  |  |  |  |
| 33 | Wood dust | 294 |  |  |  |  |  |  |  |  |  |  |  |  |  |  |  |  |  |  |  |  |  |  |  |  |  |  |  |  |  |  |  |  |  |

**Supplementary Material on Detailed Results**

As outlined in the main text, for our matched analysis Odds Ratios (ORs) and 95% confidence intervals (95% CIs) were calculated using conditional logistic regression implemented in Stata. Where the numbers of exposed cases and controls permitted the Stata Clogit command was used. Matching factors were: sex, period of birth and birth registration sub-district. ORs and 95% CIs additionally adjusted for social class (I, II, IIINM, IIIM, IV and V) were also generated. Our primary exposed population was those classified as ‘definitely’ exposed. The same analyses were repeated taking the exposed population as those with either ‘definite’ or ‘possible’ exposures. Differences between these two sets of results were minimal and all further references are to definite exposures.

In instances where there were 5 or fewer exposed cases and/or controls for any analysis, the Stata Exlogistic command was used to perform exact conditional logistic regression. Because of the small numbers involved in these analyses, it was inappropriate to adjust for social class.

Statistically significant results were defined as those where the p value was <0.05 and any significant ORs reflecting associations not previously reported in the literature were re-assessed using the Bonferroni method to allow judgements to be made on the importance of multiple significance testing. In these circumstances, simple p values are likely to suggest significance for associations that are simply due to chance. However the Bonferroni correction is likely to fail to identify genuinely significant associations. We suggest that further information and in particular additional independent studies are required to resolve such ambiguities.

Associated Tables:

Table B1: Numbers of case and Control records in different categories for birth registrations and coding of occupation and social class

Table B2: Paternal Occupational Exposures and ORs for total lymphoma

Table B3: Paternal Occupational Exposures and ORs for Hodgkin lymphoma

Table B4: Paternal Occupational Exposures and ORs for non-Hodgkin lymphoma

Table B5: Paternal Occupational Exposures and ORs for Burkitt lymphoma

**Table B1: Numbers of case and Control records in different categories for birth registrations and coding of occupation and social class**

| **Birth registrations** |  |  |
| --- | --- | --- |
|  | Cases |  |
| Born and diagnosed 1962-2010 in NRCT | 5875 |  |
| Born overseas /Adopted | 272 |  |
| Late registrations - birth record not requested | 18 |  |
| Not traced in birth registers | 171 |  |
| Eligible + birth record available | 5414 |  |
|  |  |  |
| **Occupation and social class coding** |  |  |
|  | Cases | Controls |
| Total eligible birth registrations | 5414 | 5414 |
| Missing paternal occupation | 317 | 367 |
| Unable to classify to 1980 SOC | 46 | 36 |
| Unable to convert to 1970 classification | 18 | 21 |
| Total eligible for unadjusted occupational analysis (Tables B2-B5) | 5033 | 4990 |
|  |  |  |
| Social class based upon occupation missing | 133 | 156 |
| Total eligible for adjusted occupational analyses (Tables B2-B5) | 4900 | 4834 |
|  |  |  |
| Total eligible for occupational social class analysis (Table 2) | 4918 | 4855 |

Table B2: Paternal Occupational Exposures and Odd Ratios for total lymphoma

|  | Group | exposed cases | | exposed controls | | informative pairs | OR^a^ | 95% CI | p | OR^b^ | 95% CI | p |
| --- | --- | --- | --- | --- | --- | --- | --- | --- | --- | --- | --- | --- |
|  |  | n | % | n | % | n |  |  |  |  |  |  |
| 1 | Agriculture | 119 | 2.2 | 107 | 2.0 | 196 | 1.11 | 0.84-1.47 | 0.48 | 1.10 | 0.83-1.48 | 0.51 |
| 2 | Agrochemical | 150 | 2.8 | 152 | 2.8 | 261 | 0.96 | 0.76-1.23 | 0.76 | 0.96 | 0.75-1.23 | 0.75 |
| 3 | Animals | 22 | 0.4 | 38 | 0.7 | 57 | **0.58** | 0.34-1.00 | <0.05 | **0.57** | 0.33-0.99 | <0.05 |
| 4 | Ceramics/glass | 29 | 0.5 | 14 | 0.3 | 40 | **2.33** | 1.19-4.59 | <0.05 | **2.45** | 1.22-4.95 | <0.05 |
| 5 | Coal dust | 34 | 0.6 | 47 | 0.9 | 70 | 0.67 | 0.41-1.08 | 0.10 | 0.67 | 0.41-1.10 | 0.11 |
| 6 | Construction | 374 | 6.9 | 393 | 7.3 | 661 | 0.94 | 0.81-1.10 | 0.46 | 0.93 | 0.79-1.08 | 0.34 |
| 7 | EMFs | 281 | 5.2 | 265 | 4.9 | 476 | 1.08 | 0.90-1.29 | 0.41 | 1.06 | 0.88-1.27 | 0.54 |
| 8 | Exhaust fumes | 423 | 7.8 | 432 | 8.0 | 702 | 0.95 | 0.82-1.10 | 0.50 | 0.94 | 0.81-1.10 | 0.45 |
| 9 | Fishing | 8 | 0.1 | 10 | 0.2 | 13 | 1.17 | 0.39-3.47 | 0.78 | 1.00 | .032-3.10 | 1.00 |
| 10 | Foodstuffs | 169 | 3.1 | 170 | 3.1 | 295 | 1.01 | 0.80-1.26 | 0.95 | 1.01 | 0.80-1.27 | 0.96 |
| 11 | Forces | 207 | 3.8 | 229 | 4.2 | 375 | 0.91 | 0.75-1.12 | 0.38 |  |  |  |
| 12 | Heat (prolonged exposure) | 127 | 2.3 | 129 | 2.4 | 232 | 0.97 | 0.75-1.25 | 0.79 | 0.97 | 0.74-1.25 | 0.79 |
| 13 | Hydrocarbons (inhaled) | 859 | 15.9 | 823 | 15.2 | 1186 | 1.02 | 0.91-1.15 | 0.68 | 1.02 | 0.90-1.15 | 0.79 |
| 14 | Hydrocarbons (dermal) | 446 | 8.2 | 416 | 7.7 | 682 | 1.07 | 0.92-1.25 | 0.36 | 1.06 | 0.91-1.24 | 0.46 |
| 15 | Ionising radiation | 5 | 0.1 | 4 | 0.1 | 9 | 1.25 | 0.27-6.30 | 1.00 |  |  |  |
| 16 | Lead | 157 | 2.9 | 167 | 3.1 | 292 | 0.96 | 0.76-1.21 | 0.73 | 0.95 | 0.75-1.20 | 0.68 |
| 17 | Leather | 8 | 0.1 | 14 | 0.3 | 20 | 0.54 | 0.21-1.35 | 0.19 | 0.58 | 0.23-1.48 | 0.26 |
| 18 | Medical/Health care | 94 | 1.7 | 95 | 1.8 | 165 | 1.01 | 0.75-1.37 | 0.94 | 1.03 | 0.75-1.41 | 0.87 |
| 19 | Metal | 790 | 14.6 | 757 | 14.0 | 1149 | 1.04 | 0.93-1.17 | 0.46 | 1.04 | 0.92-1.17 | 0.51 |
| 20 | Metal acid mists | 4 | 0.1 | 4 | 0.1 | 7 | 0.75 | 0.11-4.43 | 1.00 |  |  |  |
| 21 | Metal fumes | 78 | 1.4 | 59 | 1.1 | 116 | 1.42 | 0.98-2.05 | 0.07 | 1.42 | 0.98-2.07 | 0.06 |
| 22 | Metal working (oil mists) | 241 | 4.5 | 216 | 4.0 | 401 | 1.10 | 0.90-1.34 | 0.34 | 1.12 | 0.92-1.37 | 0.27 |
| 23 | Mining | 38 | 0.7 | 48 | 0.9 | 75 | 0.74 | 0.47-1.18 | 0.21 | 0.75 | 0.47-1.20 | 0.24 |
| 24 | Paints | 93 | 1.7 | 82 | 1.5 | 145 | 1.04 | 0.75-1.44 | 0.80 | 1.03 | 0.74-1.43 | 0.87 |
| 25 | Paper production | 1 | 0.0 | 1 | 0.0 | 2 | 1.00 | 0.01-78.50 | 1.00 |  |  |  |
| 26 | Plastics | 11 | 0.2 | 8 | 0.1 | 16 | 1.29 | 0.48-3.45 | 0.62 | 1.28 | 0.48-3.45 | 0.62 |
| 27 | Printing | 58 | 1.1 | 45 | 0.8 | 91 | 1.33 | 0.88-2.02 | 0.17 | 1.34 | 0.88-2.04 | 0.17 |
| 28 | Rubber | 13 | 0.2 | 12 | 0.2 | 25 | 1.08 | 0.49-2.37 | 0.84 | 1.00 | 0.45-2.22 | 1.00 |
| 29 | Social contact | 661 | 12.2 | 665 | 12.3 | 817 | 0.98 | 0.86-1.13 | 0.81 | 0.96 | 0.83-1.11 | 0.56 |
| 30 | Solvents | 164 | 3.0 | 138 | 2.5 | 231 | 1.14 | 0.88-1.47 | 0.32 | 1.14 | 0.87-1.48 | 0.34 |
| 31 | Textile dust | 86 | 1.6 | 79 | 1.5 | 142 | 1.06 | 0.76-1.47 | 0.74 | 1.07 | 0.77-1.50 | 0.68 |
| 32 | Tobacco dust | 1 | 0.0 | 0 | 0.0 | 1 |  |  |  |  |  |  |
| 33 | Wood dust | 153 | 2.8 | 141 | 2.6 | 245 | 1.09 | 0.85-1.41 | 0.48 | 1.08 | 0.84-1.39 | 0.57 |

^a^OR with only the implicit adjustment for the matching factors: sex, registration sub-district and period of registration

^b^OR additionally adjusted for occupational social class (based on ONS categories 1,2,3N,3M,4,5 defined by the father's declared occupation at the time of the child's birth)

ORs in bold indicate values which differ significantly from 1 (P<0.05)

Table B3: Paternal Occupational Exposures and Odds Ratios for Hodgkin lymphoma

|  | Group | exposed cases | | exposed controls | | Informative pairs | OR^a^ | 95% CI | p | OR^b^ | 95% CI | p |
| --- | --- | --- | --- | --- | --- | --- | --- | --- | --- | --- | --- | --- |
|  |  | n | % | n | % | n |  |  |  |  |  |  |
| 1 | Agriculture | 49 | 2.3 | 50 | 2.3 | 85 | 0.89 | 0.58-1.36 | 0.59 | 0.90 | 0.58-1.42 | 0.66 |
| 2 | Agrochemical | 63 | 2.9 | 72 | 3.3 | 116 | 0.81 | 0.56-1.17 | 0.27 | 0.80 | 0.55-1.18 | 0.27 |
| 3 | Animals | 9 | 0.4 | 18 | 0.8 | 25 | 0.56 | 0.25-1.27 | 0.17 | 0.53 | 0.22-1.25 | 0.15 |
| 4 | Ceramics/glass | 12 | 0.6 | 3 | 0.1 | 15 | **4.00** | 1.08-22.09 | 0.04 |  |  |  |
| 5 | Coal dust | 15 | 0.7 | 22 | 1.0 | 33 | 0.65 | 0.32-1.31 | 0.23 | 0.67 | 0.33-1.36 | 0.27 |
| 6 | Construction | 160 | 7.4 | 139 | 6.4 | 255 | 1.14 | 0.89-1.46 | 0.29 | 1.12 | 0.87-1.45 | 0.37 |
| 7 | EMFs | 118 | 5.4 | 106 | 4.9 | 199 | 1.07 | 0.81-1.42 | 0.62 | 1.02 | 0.77-1.35 | 0.90 |
| 8 | Exhaust fumes | 162 | 7.5 | 169 | 7.8 | 262 | 0.94 | 0.74-1.20 | 0.62 | 0.92 | 0.72-1.18 | 0.52 |
| 9 | Fishing | 5 | 0.2 | 2 | 0.1 | 4 |  |  |  |  |  |  |
| 10 | Foodstuffs | 70 | 3.2 | 70 | 3.2 | 126 | 1.07 | 0.75-1.51 | 0.72 | 1.04 | 0.73-1.49 | 0.82 |
| 11 | Forces | 86 | 4.0 | 97 | 4.5 | 156 | 0.84 | 0.61-1.14 | 0.26 |  |  |  |
| 12 | Heat (prolonged exposure) | 56 | 2.6 | 52 | 2.4 | 98 | 1.09 | 0.73-1.61 | 0.69 | 1.10 | 0.74-1.64 | 0.65 |
| 13 | Hydrocarbons (inhaled) | 342 | 15.8 | 319 | 14.7 | 454 | 1.07 | 0.89-1.29 | 0.45 | 1.04 | 0.86-1.27 | 0.66 |
| 14 | Hydrocarbons (dermal) | 176 | 8.1 | 153 | 7.0 | 255 | 1.16 | 0.91-1.49 | 0.24 | 1.13 | 0.87-1.46 | 0.36 |
| 15 | Ionising radiation | 4 | 0.2 | 0 | 0.0 | 4 |  |  |  |  |  |  |
| 16 | Lead | 55 | 2.5 | 62 | 2.9 | 105 | 0.94 | 0.64-1.38 | 0.77 | 0.97 | 0.66-1.43 | 0.89 |
| 17 | Leather | 2 | 0.1 | 5 | 0.2 | 5 | 0.25 | 0.01-2.53 | 0.38 |  |  |  |
| 18 | Medical/Health care | 39 | 1.8 | 32 | 1.5 | 59 | 1.19 | 0.71-1.98 | 0.52 | 1.30 | 0.76-2.21 | 0.34 |
| 19 | Metal | 317 | 14.6 | 285 | 13.1 | 449 | 1.17 | 0.97-1.41 | 0.10 | 1.18 | 0.97-1.43 | 0.09 |
| 20 | Metal acid mists | 0 | 0.0 | 3 | 0.1 | 3 |  |  |  |  |  |  |
| 21 | Metal fumes | 34 | 1.6 | 20 | 0.9 | 46 | **1.88** | 1.02-3.44 | 0.04 | **1.84** | 1.00-3.38 | <0.05 |
| 22 | Metal working (oil mists) | 94 | 4.3 | 82 | 3.8 | 148 | 1.18 | 0.85-1.63 | 0.32 | 1.19 | 0.85-1.66 | 0.32 |
| 23 | Mining | 17 | 0.8 | 23 | 1.1 | 36 | 0.71 | 0.37-1.39 | 0.32 | 0.74 | 0.38-1.44 | 0.37 |
| 24 | Paints | 38 | 1.8 | 35 | 1.6 | 61 | 1.03 | 0.63-1.71 | 0.90 | 1.01 | 0.60-1.69 | 0.97 |
| 25 | Paper production | 0 | 0.0 | 1 | 0.0 | 1 |  |  |  |  |  |  |
| 26 | Plastics | 7 | 0.3 | 4 | 0.2 | 8 | 1.67 | 0.32-10.73 | 0.73 |  |  |  |
| 27 | Printing | 28 | 1.3 | 19 | 0.9 | 41 | 1.41 | 0.76-2.63 | 0.28 | 1.34 | 0.71-2.50 | 0.37 |
| 28 | Rubber | 2 | 0.1 | 2 | 0.1 | 4 | 1.00 | 0.07-13.80 | 1.00 |  |  |  |
| 29 | Social contact | 267 | 12.3 | 270 | 12.4 | 321 | 0.90 | 0.72-1.12 | 0.34 | 0.91 | 0.73-1.15 | 0.45 |
| 30 | Solvents | 71 | 3.3 | 58 | 2.7 | 99 | 1.15 | 0.78-1.71 | 0.48 | 1.13 | 0.75-1.69 | 0.56 |
| 31 | Textile dust | 35 | 1.6 | 39 | 1.8 | 65 | 0.86 | 0.53-1.40 | 0.54 | 0.89 | 0.54-1.46 | 0.65 |
| 32 | Tobacco dust |  | 0.0 |  | 0.0 |  |  |  |  |  |  |  |
| 33 | Wood dust | 63 | 2.9 | 60 | 2.8 | 102 | 1.13 | 0.76-1.66 | 0.55 | 1.10 | 0.74-1.63 | 0.64 |

^a^OR with only the implicit adjustment for the matching factors: sex, registration sub-district and period of registration

^b^OR additionally adjusted for occupational social class (based on ONS categories 1,2,3N,3M,4,5 defined by the father's declared occupation at the time of the child's birth)

ORs in bold indicate values which differ significantly from 1 (P<0.05)

Table B4: Paternal Occupational Exposures and Odds Ratios for non-Hodgkin lymphoma

|  | Group | exposed cases | | exposed controls | | informative pairs | OR^a^ | 95% CI | p | OR^b^ | 95% CI | p |
| --- | --- | --- | --- | --- | --- | --- | --- | --- | --- | --- | --- | --- |
|  |  | n | % | n | % | n |  |  |  |  |  |  |
| 1 | Agriculture | 59 | 2.5 | 41 | 1.7 | 90 | 1.50 | 0.98-2.29 | 0.06 | 1.48 | 0.96-2.27 | 0.08 |
| 2 | Agrochemical | 72 | 3.0 | 60 | 2.5 | 119 | 1.20 | 0.84-1.73 | 0.31 | 1.20 | 0.83-1.73 | 0.33 |
| 3 | Animals | 12 | 0.5 | 15 | 0.6 | 26 | 0.73 | 0.34-1.60 | 0.44 | 0.75 | 0.34-1.63 | 0.47 |
| 4 | Ceramics/glass | 14 | 0.6 | 6 | 0.2 | 19 | **2.80** | 1.01-7.77 | <0.05 | **3.33** | 1.09-10.23 | 0.04 |
| 5 | Coal dust | 15 | 0.6 | 22 | 0.9 | 30 | 0.58 | 0.28-1.22 | 0.15 | 0.57 | 0.26-1.24 | 0.16 |
| 6 | Construction | 158 | 6.6 | 200 | 8.3 | 312 | **0.77** | 0.62-0.97 | 0.02 | **0.76** | 0.61-0.96 | 0.02 |
| 7 | EMFs | 120 | 5.0 | 113 | 4.7 | 203 | 1.16 | 0.88-1.53 | 0.29 | 1.16 | 0.87-1.53 | 0.31 |
| 8 | Exhaust fumes | 182 | 7.6 | 207 | 8.6 | 333 | 0.86 | 0.69-1.07 | 0.17 | 0.87 | 0.70-1.08 | 0.20 |
| 9 | Fishing | 3 | 0.1 | 7 | 0.3 | 8 | 0.60 | 0.09-3.08 | 0.73 |  |  |  |
| 10 | Foodstuffs | 70 | 2.9 | 71 | 3.0 | 118 | 1.03 | 0.72-1.48 | 0.85 | 1.07 | 0.74-1.54 | 0.73 |
| 11 | Forces | 91 | 3.8 | 102 | 4.2 | 167 | 0.92 | 0.68-1.25 | 0.59 |  |  |  |
| 12 | Heat (prolonged exposure) | 54 | 2.2 | 53 | 2.2 | 97 | 0.98 | 0.66-1.46 | 0.92 | 0.96 | 0.64-1.43 | 0.83 |
| 13 | Hydrocarbons (inhaled) | 382 | 15.9 | 390 | 16.2 | 544 | 0.94 | 0.80-1.12 | 0.49 | 0.93 | 0.78-1.11 | 0.43 |
| 14 | Hydrocarbons (dermal) | 200 | 8.3 | 204 | 8.5 | 318 | 0.98 | 0.78-1.21 | 0.82 | 0.98 | 0.78-1.22 | 0.83 |
| 15 | Ionising radiation | 0 | 0.0 | 2 | 0.1 | 2 |  |  |  |  |  |  |
| 16 | Lead | 66 | 2.7 | 82 | 3.4 | 137 | 0.80 | 0.57-1.12 | 0.20 | 0.81 | 0.58-1.14 | 0.23 |
| 17 | Leather | 6 | 0.2 | 7 | 0.3 | 13 | 0.86 | 0.29-2.55 | 0.78 | 0.87 | 0.29-2.60 | 0.81 |
| 18 | Medical/Health care | 45 | 1.9 | 44 | 1.8 | 80 | 1.05 | 0.68-1.63 | 0.82 | 0.97 | 0.62-1.52 | 0.90 |
| 19 | Metal | 352 | 14.6 | 369 | 15.3 | 531 | 0.92 | 0.78-1.10 | 0.36 | 0.91 | 0.77-1.09 | 0.32 |
| 20 | Metal acid mists | 3 | 0.1 | 1 | 0.0 | 4 | 3.00 | 0.24-157.5 | 0.63 |  |  |  |
| 21 | Metal fumes | 32 | 1.3 | 25 | 1.0 | 49 | 1.33 | 0.76-2.35 | 0.32 | 1.30 | 0.73-2.29 | 0.37 |
| 22 | Metal working (oil mists) | 112 | 4.7 | 106 | 4.4 | 194 | 1.02 | 0.77-1.35 | 0.89 | 1.04 | 0.78-1.39 | 0.78 |
| 23 | Mining | 17 | 0.7 | 22 | 0.9 | 32 | 0.68 | 0.34-1.39 | 0.29 | 0.69 | 0.33-1.43 | 0.32 |
| 24 | Paints | 38 | 1.6 | 35 | 1.5 | 59 | 0.97 | 0.58-1.61 | 0.90 | 0.98 | 0.59-1.64 | 0.95 |
| 25 | Paper production | 1 | 0.0 | 0 | 0.0 | 1 |  |  |  |  |  |  |
| 26 | Plastics | 4 | 0.2 | 3 | 0.1 | 7 | 1.33 | 0.23-9.10 | 1.00 |  |  |  |
| 27 | Printing | 25 | 1.0 | 18 | 0.7 | 40 | 1.50 | 0.80-2.82 | 0.21 | 1.52 | 0.81-2.86 | 0.20 |
| 28 | Rubber | 8 | 0.3 | 8 | 0.3 | 16 | 1.00 | 0.38-2.66 | 1.00 | 0.89 | 0.32-2.46 | 0.82 |
| 29 | Social contact | 275 | 11.4 | 286 | 11.9 | 359 | 1.01 | 0.82-1.24 | 0.96 | 0.92 | 0.74-1.14 | 0.45 |
| 30 | Solvents | 70 | 2.9 | 60 | 2.5 | 98 | 1.13 | 0.76-1.68 | 0.55 | 1.15 | 0.77-1.71 | 0.49 |
| 31 | Textile dust | 40 | 1.7 | 29 | 1.2 | 60 | 1.31 | 0.78-2.18 | 0.30 | 1.31 | 0.78-2.20 | 0.30 |
| 32 | Tobacco dust | 1 | 0.0 | 0 | 0.0 | 1 |  |  |  |  |  |  |
| 33 | Wood dust | 72 | 3.0 | 56 | 2.3 | 111 | 1.22 | 0.84-1.77 | 0.30 | 1.23 | 0.84-1.79 | 0.28 |

^a^OR with only the implicit adjustment for the matching factors: sex, registration sub-district and period of registration

^b^OR additionally adjusted for occupational social class (based on ONS categories 1,2,3N,3M,4,5 defined by the father's declared occupation at the time of the child's birth)

ORs in bold indicate values which differ significantly from 1 (P<0.05)

Table B5: Paternal Occupational Exposures and ORs for Burkitt lymphoma

|  | Group | exposed cases | | exposed controls | | informative pairs | OR^a^ | 95% CI | p | OR^b^ | 95% CI | p |
| --- | --- | --- | --- | --- | --- | --- | --- | --- | --- | --- | --- | --- |
|  |  | n | % | n | % | n |  |  |  |  |  |  |
| 1 | Agriculture | 5 | 1.0 | 9 | 1.7 | 10 | 0.67 | 0.14-2.81 | 0.75 |  |  |  |
| 2 | Agrochemical | 9 | 1.7 | 13 | 2.5 | 15 | 0.67 | 0.24-1.87 | 0.44 | 0.71 | 0.24-2.05 | 0.52 |
| 3 | Animals | 1 | 0.2 | 3 | 0.6 | 4 | 0.33 | 0.01-4.15 | 0.63 |  |  |  |
| 4 | Ceramics/glass | 3 | 0.6 | 3 | 0.6 | 4 | 1.00 | 0.14-7.10 | 1.00 |  |  |  |
| 5 | Coal dust | 1 | 0.2 | 2 | 0.4 | 3 | 0.50 | 0.01-9.60 | 1.00 |  |  |  |
| 6 | Construction | 33 | 6.3 | 31 | 5.9 | 51 | 1.13 | 0.65-1.95 | 0.68 | 1.03 | 0.58-1.80 | 0.93 |
| 7 | EMFs | 28 | 5.3 | 27 | 5.1 | 46 | 0.84 | 0.47-1.50 | 0.56 | 0.89 | 0.49-1.60 | 0.69 |
| 8 | Exhaust fumes | 44 | 8.4 | 39 | 7.4 | 64 | 1.00 | 0.61-1.63 | 1.00 | 1.09 | 0.66-1.83 | 0.73 |
| 9 | Fishing | 0 | 0.0 | 1 | 0.2 | 1 |  |  |  |  |  |  |
| 10 | Foodstuffs | 18 | 3.4 | 22 | 4.2 | 36 | 0.71 | 0.37-1.39 | 0.32 | 0.69 | 0.36-1.35 | 0.28 |
| 11 | Forces | 18 | 3.4 | 20 | 3.8 | 33 | 1.06 | 0.54-2.10 | 0.86 |  |  |  |
| 12 | Heat (prolonged exposure) | 11 | 2.1 | 11 | 2.1 | 19 | 0.90 | 0.37-2.21 | 0.82 | 0.87 | 0.35-2.14 | 0.76 |
| 13 | Hydrocarbons (inhaled) | 73 | 13.9 | 66 | 12.5 | 108 | 1.00 | 0.69-1.46 | 1.00 | 1.11 | 0.75-1.66 | 0.60 |
| 14 | Hydrocarbons (dermal) | 40 | 7.6 | 30 | 5.7 | 61 | 1.26 | 0.76-2.09 | 0.37 | 1.36 | 0.79-2.34 | 0.26 |
| 15 | Ionising radiation | 1 | 0.2 | 1 | 0.2 | 2 | 1.00 | 0.01-78.5 | 1.00 |  |  |  |
| 16 | Lead | 26 | 4.9 | 10 | 1.9 | 33 | **2.67** | 1.24-5.74 | <0.05 | **2.38** | 1.10-5.19 | 0.03 |
| 17 | Leather | 0 | 0.0 | 1 | 0.2 | 1 |  |  |  |  |  |  |
| 18 | Medical/Health care | 8 | 1.5 | 14 | 2.7 | 19 | 0.73 | 0.29-1.81 | 0.49 | 0.79 | 0.31-1.98 | 0.61 |
| 19 | Metal | 74 | 14.1 | 50 | 9.5 | 93 | 1.38 | 0.92-2.09 | 0.12 | 1.37 | 0.89-2.11 | 0.15 |
| 20 | Metal acid mists |  | 0.2 |  | 0.2 | 0 |  |  |  |  |  |  |
| 21 | Metal fumes | 9 | 1.7 | 5 | 1.0 | 10 | 2.33 | 0.53-14.0 | 0.34 |  |  |  |
| 22 | Metal working (oil mists) | 15 | 2.9 | 16 | 3.0 | 29 | 0.81 | 0.39-1.69 | 0.58 | 0.91 | 0.42-1.99 | 0.82 |
| 23 | Mining | 1 | 0.2 | 2 | 0.4 | 3 | 0.50 | 0.01-9.60 | 1.00 |  |  |  |
| 24 | Paints | 7 | 1.3 | 7 | 1.3 | 10 | 0.67 | 0.19-2.36 | 0.53 | 0.67 | 0.19-2.36 | 0.53 |
| 25 | Paper production |  | 0.2 |  | 0.2 | 0 |  |  |  |  |  |  |
| 26 | Plastics | 0 | 0.0 | 1 | 0.2 | 1 |  |  |  |  |  |  |
| 27 | Printing | 4 | 0.8 | 4 | 0.8 | 8 | 1.00 | 0.19-5.39 | 1.00 |  |  |  |
| 28 | Rubber | 1 | 0.2 | 0 | 0.0 | 1 |  |  |  |  |  |  |
| 29 | Social contact | 80 | 15.2 | 73 | 13.9 | 90 | 1.09 | 0.72-1.65 | 0.67 | 1.15 | 0.75-1.76 | 0.53 |
| 30 | Solvents | 12 | 2.3 | 11 | 2.1 | 18 | 0.80 | 0.32-2.03 | 0.64 | 0.80 | 0.31-2.02 | 0.63 |
| 31 | Textile dust | 5 | 1.0 | 8 | 1.5 | 11 | 0.57 | 0.12-2.25 | 0.55 |  |  |  |
| 32 | Tobacco dust |  | 0.2 |  | 0.2 | 0 |  |  |  |  |  |  |
| 33 | Wood dust | 13 | 2.5 | 19 | 3.6 | 25 | 0.67 | 0.30-1.48 | 0.32 | 0.57 | 0.25-1.31 | 0.18 |

^a^OR with only the implicit adjustment for the matching factors: sex, registration sub-district and period of registration

^b^OR additionally adjusted for occupational social class (based on ONS categories 1,2,3N,3M,4,5 defined by the father's declared occupation at the time of the child's birth)

ORs in bold indicate values which differ significantly from 1 (P<0.05)
